# Species-aware interpretation of vancomycin-related genomic signals in poultry-derived *Enterococcus* spp.: a confirmatory phenotyping and whole-genome sequencing framework for One Health surveillance

**DOI:** 10.3389/fvets.2026.1831285

**Published:** 2026-07-22

**Authors:** Ádám Kerek, Gergely Álmos Tornyos, Levente Hunor Husz, Eszter Kaszab, Krisztián Bányai, Krisztina Bali, Ákos Jerzsele

**Affiliations:** 1 Department of Pharmacology and Toxicology, University of Veterinary Medicine Budapest, Budapest, Hungary; 2 National Laboratory of Infectious Animal Diseases, Antimicrobial Resistance, Veterinary Public Health and Food Chain Safety, University of Veterinary Medicine Budapest, Budapest, Hungary; 3 One Health Institute, University of Debrecen, Debrecen, Hungary; 4 Department of Microbiology and Infectious Diseases, University of Veterinary Medicine, Budapest, Hungary; 5 Department of Medical Biology, Medical School, University of Pécs, Pécs, Hungary; 6 HUN-REN Veterinary Medical Research Institute, Budapest, Hungary

**Keywords:** *Enterococcus*, minimum inhibitory concentration, One Health, species identification, vancomycin, whole-genome sequencing

## Abstract

**Background:**

Poultry-associated enterococci are relevant to One Health surveillance, yet interpreting vancomycin-related genomic signals requires careful consideration of species biology and detection confidence.

**Methods:**

We analyzed broth microdilution MIC data for 218 poultry-origin *Enterococcus* isolates from an archival strain bank in Hungary and paired these data with whole-genome sequencing (WGS) for 73 isolates with linked MIC records. Vancomycin MICs for isolates previously flagged as showing elevated vancomycin MICs and/or phenotype–genotype discordant profiles were re-tested in three parallel confirmatory broth microdilution measurements using single-colony subcultures, and final confirmed MIC values are reported.

**Results:**

In the final dataset, no confirmed vancomycin MIC value exceeded 4 μg/ml. Resistome screening of the 73 genomes (CARD-based output) yielded 1,448 resistance-associated annotations (181 unique gene calls; median 12 per genome) and did not detect canonical acquired van operons consistent with *vanA, vanB, vanD, vanM*, or *vanN*. van-associated signals were limited to *vanC*-like components, *vanG*-type regulatory elements (*vanRG* and/or *vanSG*), and a single *vanW*-annotated record, highlighting the need for species-aware interpretation and locus-level validation.

**Conclusions:**

These results support a surveillance-grade workflow that anchors van-related genomic reporting to confirmatory phenotyping, genome-confirmed species assignment, and confidence-aware locus inspection to minimize false alarms and improve One Health interpretability.

## 1 Introduction

Antimicrobial resistance (AMR) is increasingly shaped by interconnected reservoirs spanning humans, food-producing animals and the environment; therefore, a One Health perspective is essential when interpreting resistance emergence, persistence and dissemination in bacteria colonizing multiple hosts and ecological niches (1, 2). *Enterococcus* spp. exemplify this challenge: they are ubiquitous gastrointestinal commensals yet also major opportunistic pathogens, with *E. faecalis* and *E. faecium* contributing substantially to healthcare-associated infections and frequently displaying multidrug resistance (3). Their persistence traits and genetic plasticity facilitate acquisition and reshuffling of antimicrobial resistance genes (ARGs) and adaptive loci, positioning enterococci as both sentinels and potential conduits of AMR across animal–food–human interfaces (1, 4–6).

Glycopeptide resistance is of particular concern. Vancomycin remains a cornerstone for severe Gram-positive infections, and vancomycin-resistant enterococci (VRE) especially *E. faecium*, continue to pose a sustained infection-control challenge, with ongoing public-health relevance documented in European surveillance (7–9). One Health risk is determined not only by clinical incidence but also by the distribution and genetic context of resistance determinants in non-human reservoirs that may support long-term maintenance and reintroduction into clinical ecosystems (2, 10).

Vancomycin resistance in enterococci is classically mediated by gene clusters that remodel peptidoglycan precursors, most importantly via acquired *vanA*- or *vanB*-type systems conferring high-level resistance, whereas *vanC*-type determinants are typically intrinsic and species-associated (notably *E. gallinarum* and *E. casseliflavus*) (7, 11–14). This distinction directly affects surveillance interpretation: species misassignment, uniform interpretive rules across non-equivalent taxa, or incomplete locus calls can lead to over- or underestimation of One Health risk (11, 12, 15, 16).

Accordingly, vancomycin phenotype–genotype concordance is not guaranteed in surveillance datasets. Apparent discrepancies can arise when van-related loci are detected in isolates with low MICs (e.g., non-expression, partial loci, or intrinsic homologs) or when MIC values fall near interpretive thresholds and calling pipelines fail to recover complete operons due to assembly fragmentation, divergent alleles below default thresholds, database/pipeline differences, or species-level misclassification (12, 13). Whole-genome sequencing (WGS) can resolve species identity, resistome content, and locus context, but requires reproducible, species-aware interpretive logic and confidence-aware reporting (identity, coverage, and locus completeness) to support surveillance-grade conclusions (17, 18).

We present a WGS-based analysis of 73 poultry-derived *Enterococcus* isolates from Hungary with linked broth microdilution MIC records, integrating resistome profiling, species-aware interrogation of vancomycin-associated determinants, and locus-context assessment to inform One Health reporting. Similar phenotype–genotype discrepancies with One Health relevance have also been described in livestock-associated staphylococci, underscoring the need for transparent, reproducible interpretation workflows when phenotypic and genomic signals diverge (19, 20). Our objective is to provide a transparent, surveillance-grade interpretation framework for vancomycin-related genomic signals in poultry-derived enterococci that anchors reporting to confirmatory phenotyping, genome-confirmed species assignment, and confidence-aware locus inspection.

## 2 Materials and methods

### 2.1 Bacterial isolates and study design

This work is based exclusively on an archival *Enterococcus* strain collection maintained at our laboratory. All isolates had been collected and cryopreserved prior to the conception of the present study and were retrieved from the strain bank for the analyses described here. Importantly, no prospective sampling, handling, or experimental procedures involving live animals were performed for this study. Accordingly, the study represents a secondary analysis of previously archived bacterial isolates, and no animal experimentation approval was required. Isolates originated from poultry production systems in Hungary, including chickens and turkeys, and associated metadata included host species, sampling site, year, and isolate identifiers.

Phenotypic susceptibility testing by broth microdilution was available for 218 isolates (MIC dataset). WGS was performed for 73 isolates (genomic dataset). Resistome profiling and vancomycin-determinant interrogation were conducted for all 73 genomes. Genotype–phenotype comparisons were performed on the subset with matched broth microdilution results (*n* = 73).

### 2.2 Antimicrobial susceptibility testing

MICs were determined by broth microdilution using twofold serial dilutions in 96-well microtiter plates with cation-adjusted Mueller–Hinton broth and standardized inoculum preparation. Plates were incubated under aerobic conditions and MIC endpoints were read as the lowest antimicrobial concentration preventing visible growth. Vancomycin MICs for isolates previously flagged as showing elevated vancomycin MICs and/or phenotype–genotype discordant profiles were re-tested in three parallel confirmatory broth microdilution measurements using freshly prepared inocula from single-colony subcultures to confirm purity. Single-colony subcultures were used to confirm culture purity and to minimize the risk of mixed cultures or isolate-level handling errors. The vancomycin MIC values reported in this manuscript reflect the final confirmatory testing results. *E. faecalis* ATCC 29212 was included as the quality-control strain.

The antimicrobial panel comprised 19 agents: amoxicillin, amoxicillin–clavulanic acid, azithromycin, doxycycline, oxytetracycline, enrofloxacin, florfenicol, imipenem, vancomycin, ceftriaxone, neomycin, spectinomycin, tilmicosin, tylosin, tiamulin, lincomycin, colistin, linezolid, and potentiated sulfonamide. Clinical breakpoints and epidemiological cut-off values (ECOFFs) used for interpretive categorization were compiled *a priori* from internationally recognized interpretive standards and databases, with species applicability considered where available. Agents without predefined interpretive criteria were reported descriptively and were not included in categorical susceptibility summaries. For vancomycin, CLSI-derived *Enterococcus* interpretive categories were applied, with isolates categorized as susceptible when MIC values did not exceed 4 μg/ml; no isolate met the intermediate or resistant categories after confirmatory testing.

### 2.3 DNA extraction, library preparation, and sequencing

Genomic DNA was extracted from bacterial suspensions using the Zymo Quick-DNA Fungal/Bacterial kit (Zymo Research, Irvine, CA, USA). Libraries were prepared with the Illumina Nextera XT DNA Library Preparation Kit (Illumina, San Diego, USA) and quantified by qPCR prior to pooling (21, 22). Sequencing was performed on an Illumina NextSeq 500 platform using single-end Illumina reads (R1 only).

### 2.4 Read preprocessing and *de novo* assembly

Raw read quality was assessed using FastQC v0.12.1 (https://github.com/s-andrews/FastQC/releases). Adapter trimming and quality filtering were performed using fastp v1.0.1 (https://github.com/OpenGene/fastp). *De novo* assemblies were generated from the cleaned single-end reads (R1 only) using SPAdes v4.2.0 in isolate genome mode with the –careful option (https://github.com/ablab/spades). Contigs shorter than 200 bp were removed prior to downstream analyses and deposition. Assembly quality metrics were evaluated using QUAST v5.3 (https://github.com/ablab/quast/releases). For all downstream AMR calling and locus-level analyses, only the final SPAdes assembly per isolate was used.

### 2.5 Species confirmation and genome-level quality checks

Initial taxonomic classification was performed using Kraken2 (v2.17.1; https://github.com/DerrickWood/kraken2), and KrakenTools (v1.2.1; https://github.com/jenniferlu717/KrakenTools) was used for downstream report parsing and summarization. Genome-level quality control was supported by CheckM v1.2.4 (available at: https://github.com/Ecogenomics/CheckM). Because species identity is critical for species-intrinsic vancomycin signals (e.g., *vanC*-associated taxa) and for interpreting discordant phenotype–genotype patterns, ANI (FastANI) confirmation was performed for all 73 genomes.

### 2.6 Resistome profiling and vancomycin determinant interrogation

Primary resistome screening was performed using RGI v5.1.0 with CARD. ABRicate-based CARD screening was used as an independent cross-check for selected resistance-associated calls. For primary resistome summaries, gene presence/absence was recorded based on sequence identity and gene coverage metrics; ambiguous or borderline hits were retained for manual review rather than discarded, to avoid false negatives in mechanistically important loci.

Vancomycin-associated determinants were interrogated using a multi-evidence strategy to minimize database- and threshold-dependent miscalls: (i) CARD/RGI calls as above, (ii) independent cross-checking with AMRFinderPlus and ResFinder on assembled genomes, and (iii) targeted BLAST searches against curated reference sequences representing canonical acquired van operons (*vanA, vanB, vanD, vanM, vanN*) and vanC-associated components.

For targeted BLAST, two reporting tiers were applied and explicitly tracked: a stringent tier (≥95% nucleotide identity and ≥90% query coverage) for primary calls, and a relaxed tier (≥80% identity and ≥50% coverage) to flag divergent or fragmented candidates requiring manual inspection. Read mapping was performed for all 73 isolates using the available raw FASTQ files (single-end R1) against the curated canonical van operon references to evaluate whether apparent absence could reflect assembly fragmentation.

AMRFinderPlus, ResFinder, and targeted BLAST analyses were used as confirmatory screens for vancomycin-associated loci. Primary interpretation was based on RGI/CARD outputs, while discordant or borderline calls were resolved by manual inspection of sequence identity, gene coverage, contig context, operon/module completeness, and read-mapping support where available. Software versions, database access dates, analytical thresholds, and conflict-resolution rules are summarized in Supplementary Table S3.

### 2.7 Mobile genetic elements and genomic context analyses

Mobile genetic elements (MGEs) were detected using MobileElementFinder v1.0.3 (23) on the final SPAdes assemblies. For resistome-level context, ARG coordinates were evaluated for proximity to MGE features, and a ±10 kb window was used as the operational definition for co-localization.

For vancomycin-associated loci, gene-neighborhood analyses were conducted by extracting and annotating ±10 kb regions around van hits and selected candidate regions requiring manual review, documenting the presence of mobility signatures (e.g., transposases, integrases) and co-localized ARGs, to support One Health–relevant interpretation of potential mobility.

### 2.8 Statistical analysis and visualization

All genomes (*n* = 73) were included in presence/absence summaries of van-associated signals and the broader resistome. For the WGS subset, marker–phenotype associations for selected antimicrobial classes (restricted to agents with predefined interpretive criteria) were evaluated using two-sided Fisher's exact tests on 2 × 2 contingency tables. We report odds ratios with 95% confidence intervals together with exact *p*-values; for multiple comparisons across drug–marker tests, *p*-values were adjusted using Benjamini–Hochberg false discovery rate control and are reported as q-values. Species-stratified analyses were performed only where sample sizes and cell counts allowed; otherwise, analyses are presented as exploratory. All analyses and visualizations were performed in R v4.1.0.

## 3 Results

### 3.1 Study datasets and vancomycin MIC profile

Phenotypic broth microdilution MIC data were available for 218 *Enterococcus* isolates. WGS was performed for 73 isolates from an archival strain bank, and all sequenced isolates had linked broth microdilution MIC records (*n* = 73).

Across the MIC dataset (*n* = 218), isolates originated from poultry production systems and included both chickens (126/218, 57.8%) and turkeys (92/218, 42.2%). Species identity for the WGS subset (*n* = 73) was confirmed using genome-based approaches (Kraken2 and ANI).

**Figure 1:**
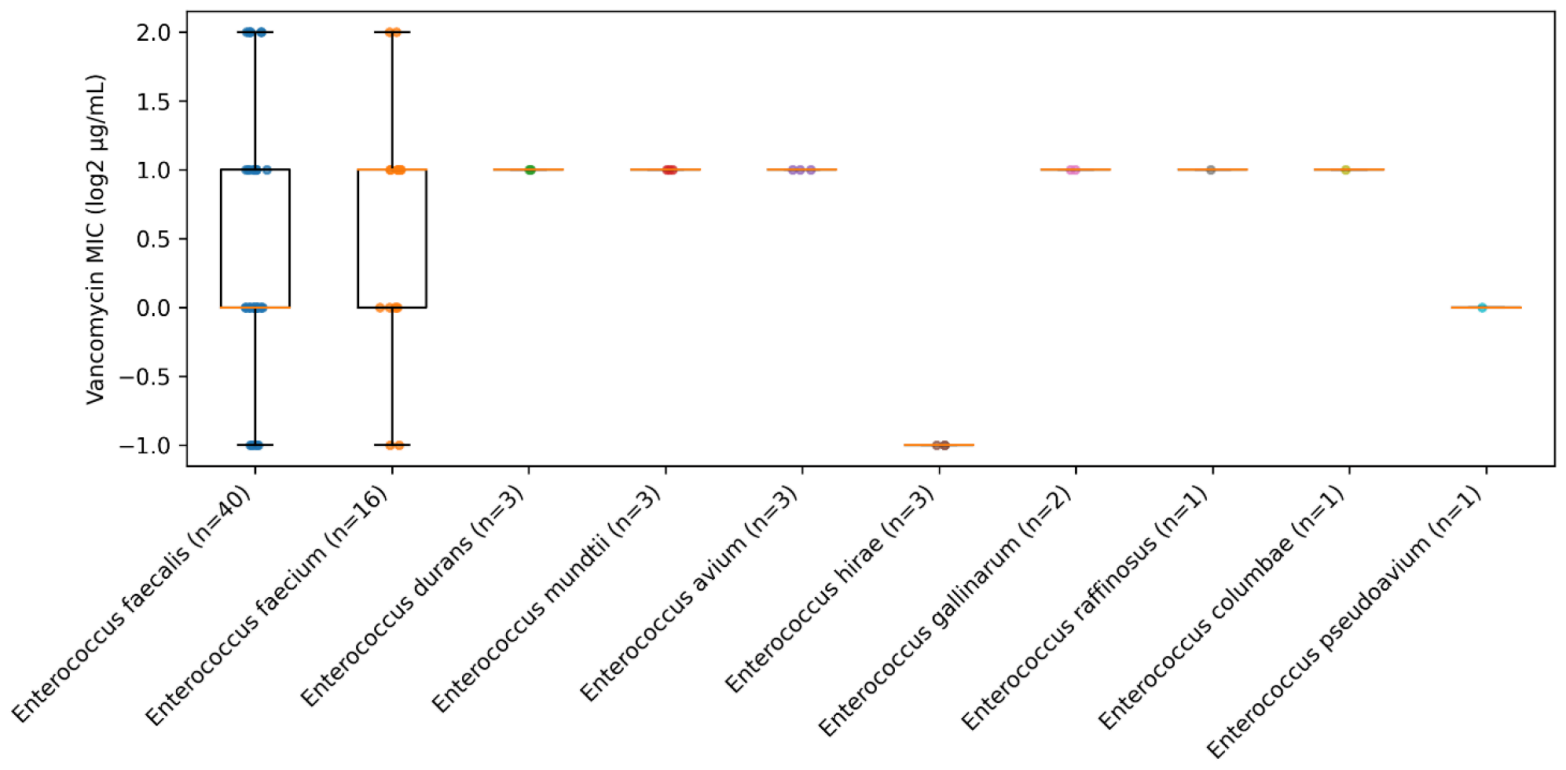
Vancomycin minimum inhibitory concentration (MIC) distributions by genome-confirmed *Enterococcus* species in the whole-genome sequencing (WGS) subset (*n* = 73). Broth microdilution vancomycin MIC values are shown by species with individual isolate-level data points; values are displayed on a log_2_-transformed MIC scale.

Following three parallel confirmatory broth microdilution measurements, no final confirmed vancomycin MIC value exceeded 4 μg/ml in any isolate (Supplementary Table S1). Accordingly, under the CLSI-derived interpretive criteria applied in this study, none of the 218 isolates met the definition of vancomycin-intermediate or vancomycin-resistant *Enterococcus* (Table 1). Vancomycin MIC distributions by genome-confirmed species in the WGS subset are shown in Figure 1.

**Table 1:** Phenotypic susceptibility category distributions across antimicrobial agents with predefined interpretive criteria in the full broth microdilution minimum inhibitory concentration (MIC) dataset (*n* = 218).

Antimicrobial agent	S, *n* (%)	I, *n* (%)	R, *n* (%)
Amoxicillin	195 (89.4%)	0 (0.0%)	23 (10.6%)
Amoxicillin–clavulanic acid^*^	218 (100.0%)	0 (0.0%)	0 (0.0%)
Azithromycin	73 (33.5%)	9 (4.1%)	136 (62.4%)
Doxycycline	75 (34.4%)	28 (12.8%)	115 (52.8%)
Enrofloxacin	100 (45.9%)	26 (11.9%)	92 (42.2%)
Florfenicol	11 (5.0%)	75 (34.4%)	132 (60.6%)
Imipenem	202 (92.7%)	0 (0.0%)	16 (7.3%)
Lincomycin	4 (1.8%)	2 (0.9%)	212 (97.2%)
Oxytetracycline	63 (28.9%)	4 (1.8%)	151 (69.3%)
Trimethoprim–sulfamethoxazole^**^	11 (5.0%)	34 (15.6%)	173 (79.4%)
Tylosin	69 (31.7%)	2 (0.9%)	147 (67.4%)
Vancomycin	218 (100.0%)	0 (0.0%)	0 (0.0%)
Linezolid	161 (73.9%)	41 (18.8%)	16 (7.3%)

* Amoxicillin–clavulanic acid was tested at a 2:1 ratio; ^**^trimethoprim–sulfamethoxazole was tested at a 1:19 ratio. Interpretive categories were assigned using the predefined criteria described in the Methods. S, susceptible; I, intermediate; R, resistant.

### 3.2 Broader antimicrobial susceptibility and ECOFF context

Broth microdilution MICs were determined for a 19-agent panel. Clinical interpretive categorization was applied only for agents with predefined criteria in this study (13/19 agents).

**Figure 2:**
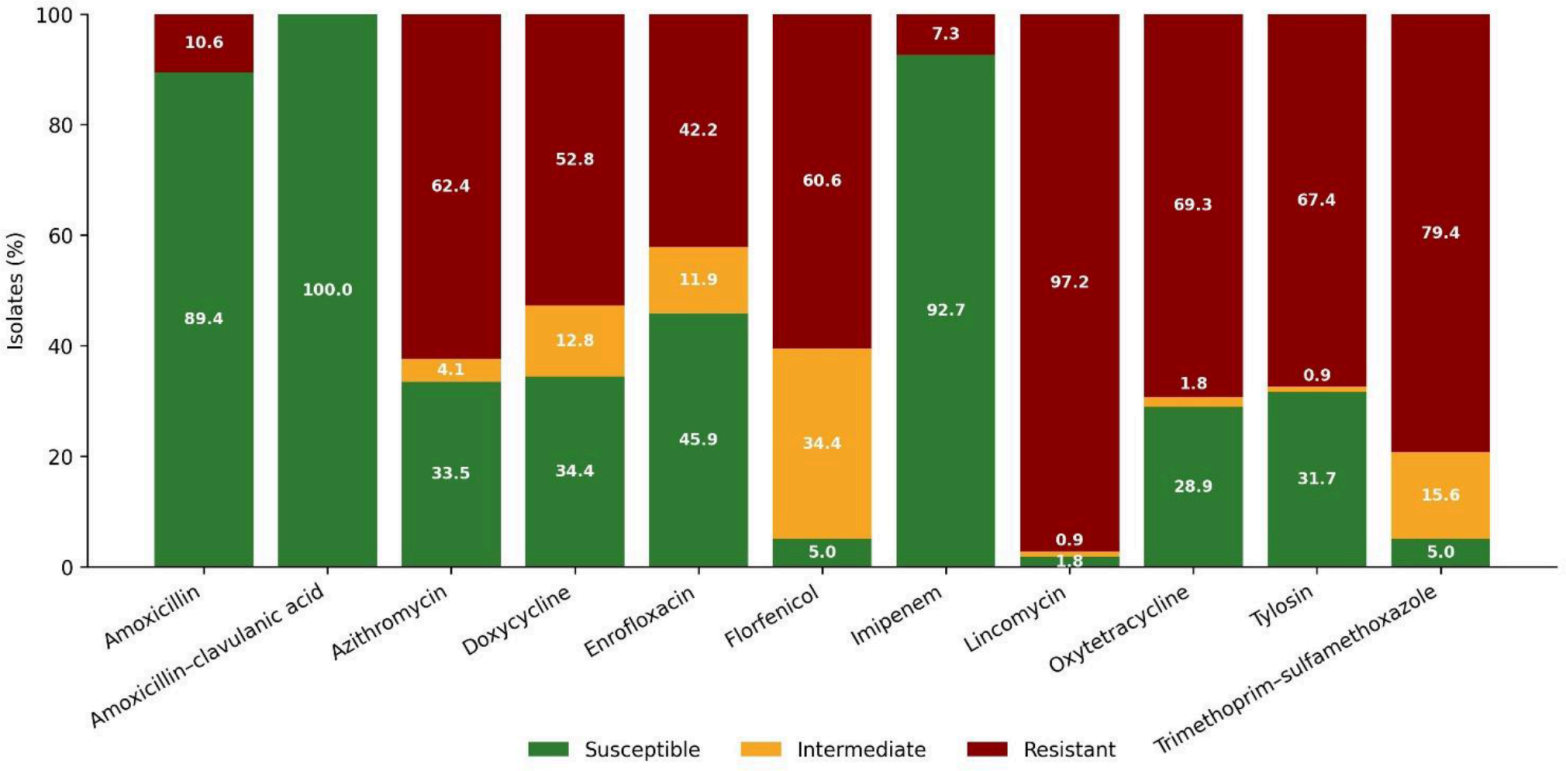
Susceptibility category profiles for 11 antimicrobial agents with predefined interpretive criteria in the full broth microdilution minimum inhibitory concentration (MIC) dataset (*n* = 218). Stacked bars show the percentage of isolates categorized as susceptible, intermediate, or resistant for each antimicrobial agent. Vancomycin and linezolid are intentionally omitted from this overview figure because vancomycin is addressed separately in the glycopeptide-focused analysis and linezolid is not part of the broader comparative susceptibility visualization. Numeric labels indicate category percentages.

Across the 13 agents with interpretive criteria, the full susceptibility category distribution is summarized in Table 1, while Figure 2 visualizes the 11 non-glycopeptide, non-oxazolidinone agents included in the broader susceptibility overview. Resistance was most frequent against lincomycin (212/218, 97.2% resistant) and trimethoprim–sulfamethoxazole (173/218, 79.4% resistant), followed by macrolides (azithromycin: 136/218, 62.4% resistant; tylosin: 147/218, 67.4% resistant) and tetracyclines (doxycycline: 115/218, 52.8% resistant; oxytetracycline: 151/218, 69.3% resistant). Florfenicol showed predominant non-susceptibility (132/218, 60.6% resistant; 75/218, 34.4% intermediate). In contrast, beta-lactam activity was largely preserved under the applied criteria (amoxicillin: 195/218, 89.4% susceptible; amoxicillin–clavulanic acid: 218/218, 100% susceptible), imipenem (202/218, 92.7% susceptible) and linezolid remained predominantly active (161/218, 73.9% susceptible). Enrofloxacin susceptibility was moderate (100/218, 45.9% susceptible; 92/218, 42.2% resistant).

Using the predefined ECOFF-based thresholds, non-wild-type proportions were summarized descriptively for selected agents. The highest proportions at/above ECOFF were observed for doxycycline (≥0.5 μg/ml: 204/218, 93.6%), neomycin (≥64 μg/ml: 175/218, 80.3%), and oxytetracycline (≥8 μg/ml: 155/218, 71.1%), whereas imipenem had the lowest fraction at/above ECOFF (≥4 μg/ml: 32/218, 14.7%).

### 3.3 Resistome overview in the 73 WGS genomes

Resistome profiling of the 73 genomes identified 1,448 resistance-associated annotations corresponding to 181 unique resistance-associated gene calls (Supplementary Table S2). The number of unique resistance-associated gene calls detected per genome was heterogeneous (median 12; IQR 10–18; range 5–76), consistent with a shared baseline of intrinsic/efflux-linked loci and a variably distributed accessory component. The most prevalent determinants included *efrA* (66/73, 90.4%), *efrB* (57/73, 78.1%), *lsaA* (57/73, 78.1%), *dfrE* (57/73, 78.1%), and *emeA* (50/73, 68.5%). Beyond this core background, acquired class-associated markers were also common, including tetracycline determinants (e.g*., tet(L)* 34/73 and *tet(M)* 24/73), macrolide-associated genes (e.g., *ermB* 24/73 and *msrC* 13/73), aminoglycoside-modifying enzymes (e.g., *AAC(6*′*)-Ii* 20/73), and lincosamide nucleotidyltransferases (e.g., *lnuB* 11/73).

### 3.4 Vancomycin-related genomic signals in the WGS subset

**Figure 3:**
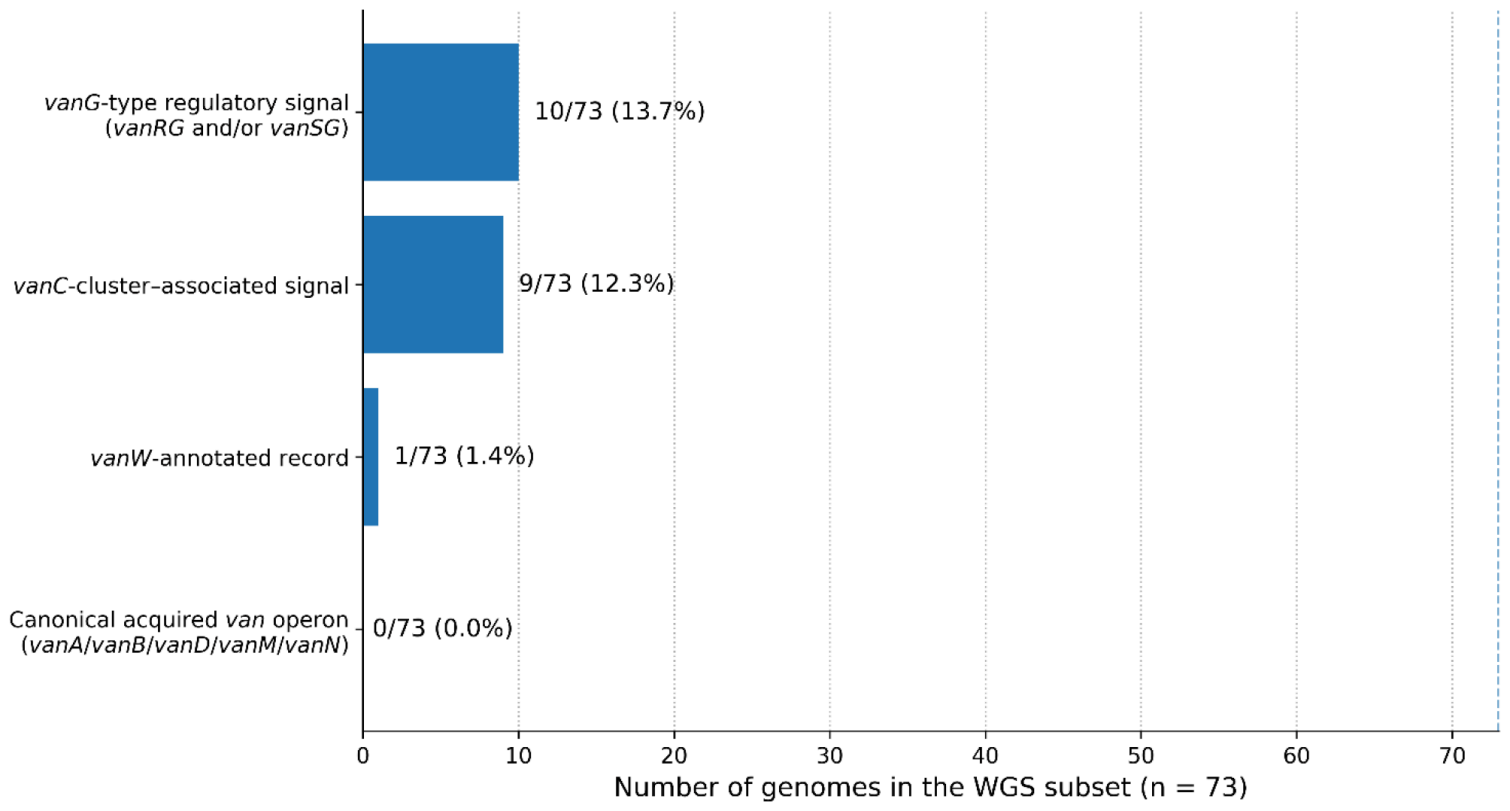
Vancomycin-associated genomic signals in the whole-genome sequencing (WGS) subset (*n* = 73). Bars show the number and proportion of genomes in which each vancomycin-associated signal category was detected in the primary resistome output. No canonical acquired van operon consistent with *vanA, vanB, vanD, vanM*, or *vanN* was detected. Detected signals were limited to *vanC*-cluster–associated components, *vanG*-type regulatory homologs (*vanRG* and/or *vanSG*), and a single *vanW*-annotated record. Categories are not mutually exclusive and should be interpreted as genomic signal classes requiring species-aware and locus-level validation rather than as direct evidence of phenotypic vancomycin resistance.

In the primary CARD-based resistome output, no canonical acquired van operons consistent with vanA, vanB, vanD, vanM, or vanN were detected among the 73 genomes. *Van*-associated signals were limited to *vanC*-cluster–associated components, *vanG*-type regulatory homologs (*vanRG* and/or *vanSG*), and a single *vanW*-annotated record (Supplementary Table S3; Figure 3).

Following three parallel confirmatory broth microdilution measurements, vancomycin MIC values in the final dataset did not exceed 4 μg/ml. Accordingly, van-associated calls in the WGS subset are interpreted as species- and context-dependent signals that require species-aware interpretation and confidence-aware locus inspection rather than as evidence of high-level glycopeptide non-susceptibility.

### 3.5 Genotype–phenotype context across selected drug classes in the WGS subset

To contextualize the vancomycin-focused analyses, we evaluated marker-level genotype–phenotype coherence for selected antimicrobial classes in the WGS subset (*n* = 73) using the predefined interpretive criteria (Table 2). At the class level, concordance was most evident for tetracyclines: at least one *tet* determinant was present in 56/73 genomes (76.7%) and was enriched among oxytetracycline-resistant isolates (50/57, 87.7%; Fisher's exact test *p* = 1.29 × 10^−4^). In contrast, macrolide-associated markers (erm^*^/msr^*^) showed only partial concordance with resistance phenotypes, and canonical phenicol markers (*fexA*/cat^*^) were infrequent relative to the observed florfenicol non-susceptibility. For potentiated sulfonamide and lincomycin, marker-based inference was constrained by widespread determinant presence and/or near-saturation of the resistant phenotype (Table 2). These observations support the interpretation that vancomycin-related genomic signals require a distinct interpretive framework, because isolated van-associated calls may occur without confirmed elevated MICs and therefore require targeted, locus-level follow-up.

**Table 2:** Marker–phenotype associations for selected antimicrobial classes in the whole-genome sequencing (WGS) subset (*n* = 73).

Antimicrobial phenotype	Gene marker	R & marker+	R & marker–	Non-R & marker+	Non-R & marker–	OR (95% CI)	*p*-value	q-value
Doxycycline (R vs non-R)	Any *tet* gene	39	7	17	10	3.28 (1.07–10.06)	0.0458	0.183
Oxytetracycline (R vs non-R)	Any *tet* gene	50	7	6	10	11.90 (3.30–43.01)	0.000129	0.001032
Azithromycin (R vs non-R)	Any *erm/msr* gene	29	27	6	11	1.97 (0.64–6.06)	0.277	0.443
Tylosin (R vs non-R)	Any *erm/msr* gene	28	24	7	14	2.33 (0.81–6.72)	0.129	0.344
Florfenicol (R vs non-R)	Any *fex/cat* gene	7	36	3	27	1.75 (0.41–7.40)	0.510	0.510
Potentiated sulfonamide (R vs non-R)	Any *dfr* gene	50	8	15	0	0.19 (0.01–3.51)	0.193	0.386
Lincomycin (R vs non-R)	Any *lnu* gene	17	54	1	1	0.31 (0.02–5.31)	0.435	0.497
Lincomycin (R vs non-R)	*lsaA*	56	15	1	1	3.73 (0.22–63.25)	0.393	0.497

Non-R includes susceptible and intermediate isolates. Marker positivity was defined based on the presence of at least one gene belonging to the indicated marker group in the primary resistome output. ORs and 95% confidence intervals were calculated with a 0.5 continuity correction where zero-cell counts were present. These analyses were exploratory and were used to contextualize genotype–phenotype coherence rather than to infer causal associations. Associations were evaluated using two-sided Fisher's exact tests; odds ratios (ORs) are reported with 95% confidence intervals, and *p*-values were adjusted using the Benjamini–Hochberg false discovery rate method. R: resistant.

### 3.6 Targeted locus-level validation and genomic context

To move beyond single-gene presence/absence and to support species-aware interpretation of van-associated calls, we performed targeted locus-level inspection of van-signal regions.

**Figure 4:**
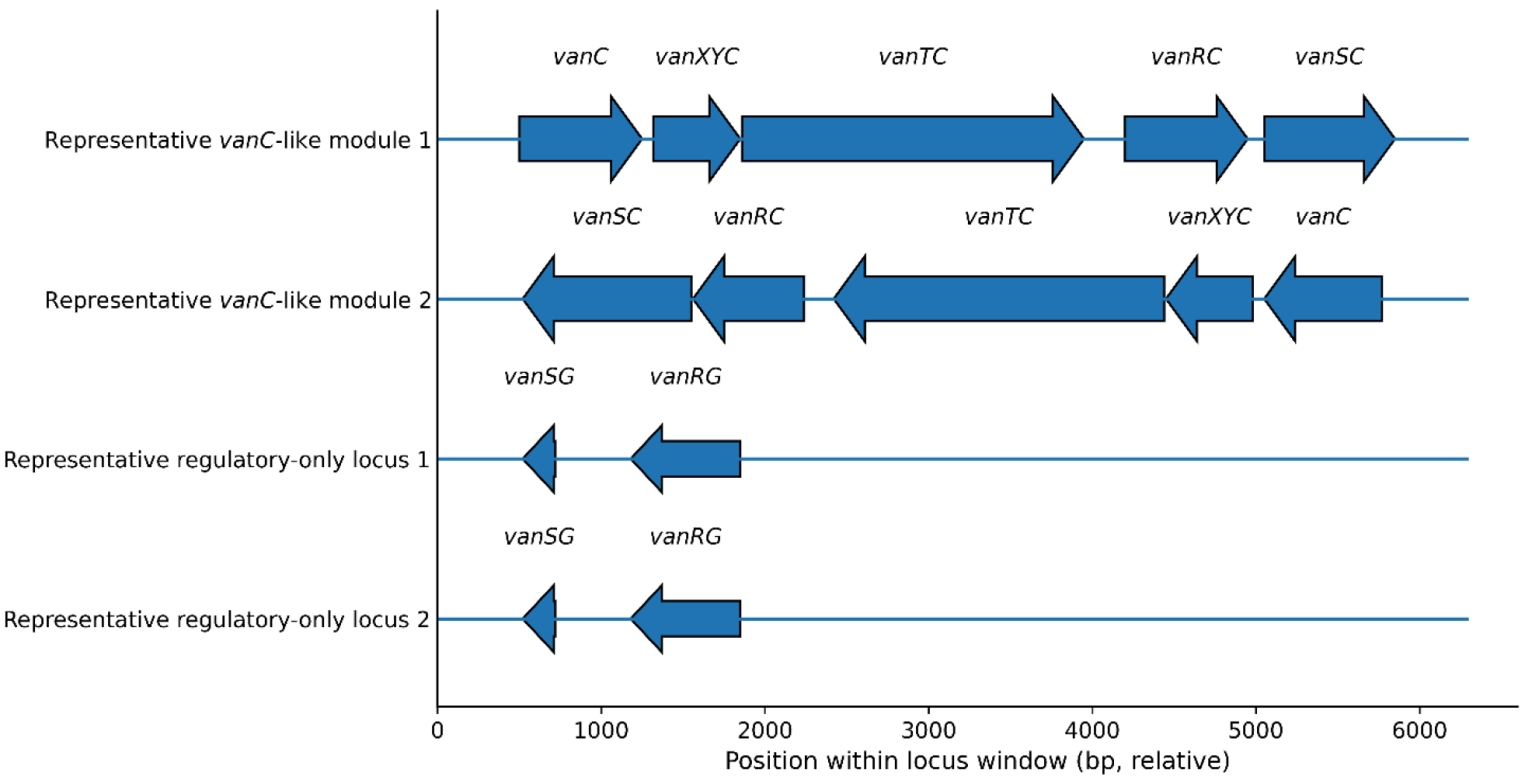
Representative genomic neighborhoods of vancomycin-associated calls detected in the whole-genome sequencing (WGS) subset. Contig-level schematic maps show local regions surrounding selected van-associated calls recovered from the primary resistome output, including *vanC*-like modules and regulatory-only *vanG*-type signals. Arrows indicate annotated vancomycin-associated genes and their relative orientation within the inspected neighborhood. These maps are intended to visualize local locus organization; they do not establish plasmid or chromosomal localization, horizontal transfer, operon functionality, or phenotypic vancomycin resistance without additional long-read or experimental validation.

Among the nine genomes with *vanC*-cluster–associated signals, six carried a complete *vanC*-type module (*vanC* plus four associated *vanC*-cluster components, independent of naming convention in the output), while the remaining genomes showed partial complements consistent with fragmented detection and/or assembly-related incompleteness. Importantly, *vanC*-type modules were detected despite confirmed vancomycin MIC values remaining within the susceptible range in the final dataset. This supports the interpretation that *vanC*-like module detection alone should not be used as evidence of clinically relevant glycopeptide non-susceptibility without species confirmation, locus-level validation, and phenotypic correlation. In representative genomes with complete positional information, the *vanC*-type components mapped as a compact ~5.3 kb cluster on a single contig and no additional resistance genes were detected within ±10 kb, supporting an intrinsic-like local locus architecture rather than a composite, multi-resistance island within the inspected ±10 kb neighborhood (Figure 4). Given that *vanC*-type determinants require species-aware interpretation, the occurrence of *vanC*-like modules in non-canonical species assignments was treated as an interpretation flag that motivates confirmatory taxonomic validation and careful locus review rather than immediate inference of intrinsic *vanC*-type resistance.

Regulatory hits annotated as *vanG*-cluster variants (*vanRG/vanSG*) were detected in ten genomes. In all genomes carrying both regulators (*n* = 8), *vanSG* and *vanRG* were co-localized on the same contig with a conserved spacing (~423 bp) and lacked accompanying *vanG* structural genes in the resistome output. The local ±10 kb neighborhoods of these regulator loci did not contain additional resistance genes in representative contigs, supporting interpretation as isolated regulatory homologs and/or truncated remnants rather than evidence of an acquired, functional *vanG* operon. A single *vanW* call annotated in a *vanB*-cluster context co-occurred with *vanC*-cluster components but was not accompanied by canonically acquired van operon genes in the available output and was therefore handled as a locus-level flag requiring confirmatory interrogation rather than as standalone evidence of an acquired *vanB*-type determinant.

Collectively, this targeted locus-level interrogation supports three conclusions: (i) canonical acquired van operons were not recovered in the available resistome calls, (ii) the van-associated signals that were recovered largely reflected vanC-type modules and isolated vanG-type regulatory homologs with strong dependence on species-aware interpretation and locus completeness, and (iii) the final confirmed phenotypes did not support high-level glycopeptide non-susceptibility, emphasizing the importance of confirmatory phenotyping before assigning One Health significance to van-related genomic signals.

## 4 Discussion

We combined broth microdilution MIC profiles from a poultry-derived *Enterococcus* collection with WGS-based resistome outputs to evaluate how vancomycin-related genomic signals should be interpreted in a One Health surveillance context. Following three parallel confirmatory measurements, vancomycin MIC values in the final dataset did not exceed 4 μg/ml. Accordingly, the manuscript focuses on (i) species-aware interpretation of van-related calls observed in genome data and (ii) a reproducible workflow that prioritizes robust phenotyping and transparent locus-level evidence when genomic signals are detected.

Enterococci are ubiquitous intestinal commensals and important opportunistic pathogens, with *E. faecium* and *E. faecalis* representing the most clinically significant species in human medicine (24). Mechanistically, vancomycin resistance is classically mediated by gene clusters that remodel peptidoglycan precursor termini, most commonly through D-Ala–D-Lac formation (*vanA*- and *vanB*-type systems), reducing glycopeptide binding and increasing MICs. In addition, certain species carry intrinsic *vanC*-type determinants that are generally interpreted as low-level glycopeptide resistance and are species-restricted (7, 11, 13, 14). These categories are not interchangeable for One Health inference, acquired *vanA/vanB*-type clusters have clearer links to mobilization and onward dissemination, whereas *vanC*-type signals primarily demand accurate species identification and cautious interpretation.

The ecology of glycopeptide resistance in food animals has historically been influenced by antimicrobial selection pressures, including glycopeptide use in some production systems. Although vancomycin itself is not a poultry-production driver, acquired glycopeptide resistance determinants remain high-consequence targets for One Health surveillance because of their potential clinical relevance and mobility (25–28). In the present dataset, however, three parallel confirmatory broth microdilution measurements did not confirm elevated vancomycin MICs above 4 μg/ml. Therefore, the central interpretation is not the detection of phenotypic VRE, but the need to prevent overinterpretation of van-associated genomic signals when they are not supported by confirmatory phenotype and operon-level evidence.

Several non-mutually exclusive explanations may account for apparent discrepancies between vancomycin-related genomic signals and confirmed low vancomycin MICs. The first possibility is technical under-detection. Assembly fragmentation across a locus, stringent identity/coverage thresholds, incomplete database representation for divergent alleles, or annotation collapse of operon components can all lead to false-negative calls, particularly for loci on mobile elements that exhibit structural variation. AMR calls can vary across databases and thresholds; confidence metrics are therefore essential for operon-scale mechanisms (29, 30). A second possibility is biological complexity beyond gene presence, including silent/disrupted loci and regulatory states that uncouple genotype from categorical phenotype. In the opposite direction of discordance, *vanA*-positive but vancomycin-susceptible (“vancomycin-variable”) enterococci have become a recognized diagnostic and surveillance challenge (31). In the present dataset, the main interpretive challenge was instead the presence of van-associated genomic signals in isolates with confirmed low vancomycin MICs. This pattern is best handled by confidence-aware locus inspection rather than by assigning categorical resistance based on isolated gene annotations.

As an internal validity check, marker–phenotype associations for other antimicrobial classes showed variable but interpretable patterns, particularly for tetracycline resistance. By contrast, vancomycin-related interpretation depended less on categorical resistance and more on whether isolated van-associated calls represented complete, species-consistent, biologically meaningful loci. These findings support treating glycopeptide-related genomic signals as a distinct interpretive category in surveillance workflows.

The *vanC*-like signals underscore why species-aware interpretation is indispensable. *vanC*-type determinants are generally species-restricted, chromosomally encoded systems linked to intrinsic glycopeptide resistance (7, 32), yet in our WGS subset *vanC*-cluster components were called in isolates not classically considered intrinsic *vanC* carriers. Accordingly, these calls should be reported as interpretation flags that prompt genome-based species verification and confidence-aware locus validation (identity/coverage and operon completeness), particularly if future isolates show elevated vancomycin MICs or if van-associated calls occur in unexpected species backgrounds.

Regulatory-only van annotations (*vanRG* and *vanSG* without an operon-scale call) likewise require caution. In our dataset, these calls occurred across MIC categories and did not map cleanly to phenotype. Mechanistically, vanR/vanS two-component systems are meaningful only in the context of a compatible effector module; isolated regulatory homologs or partial matches may represent non-functional remnants, distant homologs or database-driven misassignments rather than bona fide glycopeptide resistance circuitry. Accordingly, regulatory-only calls should not be used as stand-alone evidence of vancomycin resistance potential, particularly when identity/coverage suggests partial or low-confidence matches.

From a One Health surveillance standpoint, any elevated vancomycin MIC or any high-confidence acquired van operon signal should trigger systematic, species-aware genomic follow-up. Conversely, isolated vanC-like or regulatory-only calls in isolates with low confirmed MICs should be reported cautiously and should not be interpreted as evidence of phenotypic VRE without locus-level support.

This study has limitations. The WGS subset was resistance-enriched by design and is therefore not intended to estimate the prevalence of vancomycin resistance in Hungarian poultry production. The resistome analysis relied on short-read assemblies and database-derived annotation output; long-read sequencing and complete plasmid reconstruction were not performed, limiting structural resolution of mobile genetic elements and operon completeness assessment. Finally, gene presence does not imply expression, and isolated van-associated annotations require targeted confirmation if future datasets identify elevated MICs, unexpected species associations, or incomplete operon structures.

Overall, these findings emphasize that interpreting glycopeptide resistance in animal-associated enterococci requires species-aware, confidence-aware genomic validation rather than binary resistome outputs.

## 5 Conclusion

In this poultry-derived *Enterococcus* collection, final confirmed vancomycin MIC values did not exceed 4 μg/ml. In the WGS subset, canonical acquired van operons (*vanA/vanB/vanD/vanM/vanN*) were not detected, while van-associated signals were limited to *vanC*-like components and regulatory elements that require species-aware interpretation.

Collectively, these findings support a surveillance-grade interpretation workflow that anchors reporting to confirmatory phenotyping, genome-confirmed species assignment, and confidence-aware locus inspection when van-related genomic signals are observed.

## Data Availability

The datasets analyzed in this study are available in NCBI under BioProject accession PRJNA1474297 (https://www.ncbi.nlm.nih.gov/bioproject/PRJNA1474297). Genome assemblies (contig FASTA files) generated in this study have been deposited under the same BioProject. BioSample records are available under accession numbers SAMN56516723–SAMN56516795. Raw sequencing reads (single-end Illumina FASTQ) have been deposited in the NCBI Sequence Read Archive under BioProject PRJNA1474297, with SRA accession numbers SRR56516723–SRR56516795. All other data supporting the findings of this study are included in the article and its Supplementary material.

## References

[1] Economou V Gousia P. Agriculture and food animals as a source of antimicrobial-resistant bacteria. IDR. (2015) 8:49–61. 10.2147/IDR.S55778 PMC4388096 25878509

[2] McEwen SA Collignon PJ. Antimicrobial Resistance: a One Health Perspective. Microbiol Spectr. (2018) 6. doi: 10.1128/microbiolspec.ARBA-0009-2017 29600770 PMC11633550

[3] Fisher K Phillips C. The ecology, epidemiology and virulence of *Enterococcus*. Microbiology. (2009) 155:1749–57. doi: 10.1099/mic.0.026385-0 19383684

[4] Byappanahalli MN Nevers MB Korajkic A Staley ZR Harwood VJ. Enterococci in the environment. Microbiol Mol Biol Rev. (2012) 76:685–706. doi: 10.1128/MMBR.00023-12 23204362 PMC3510518

[5] Shiadeh SMJ Pormohammad A Hashemi A Lak P. Global prevalence of antibiotic resistance in blood-isolated *Enterococcus faecalis* and *Enterococcus faecium*: a systematic review and meta-analysis. IDR. (2019) 12:2713–25. 31564921 10.2147/IDR.S206084 PMC6731464

[6] Farkas Z Csorba S Vribék K Süth M Czudor Z Tényi Á . Establishing a data infrastructure system for veterinary public health and food chain safety data through the development of a repositioning platform. Magyar Állatorvosok Lapja. (2025) 147:621–34. doi: 10.56385/magyallorv.2025.10.621-634

[7] Arthur M Courvalin P. Genetics and mechanisms of glycopeptide resistance in enterococci. Antimicrob Agents Chemother. (1993) 37:1563–71. doi: 10.1128/AAC.37.8.1563 8215264 PMC188020

[8] O'Driscoll T Crank CW. Vancomycin-resistant enterococcal infections: epidemiology, clinical manifestations, and optimal management. IDR. (2015) 8:217–30. 26244026 10.2147/IDR.S54125 PMC4521680

[9] Almeida-Santos AC Novais C Peixe L Freitas AR. Vancomycin-resistant *Enterococcus faecium*: A current perspective on resilience, adaptation, and the urgent need for novel strategies. J Glob Antimicrob Resist. (2025) 41:233–52. doi: 10.1016/j.jgar.2025.01.016 39880121

[10] Benmazouz I Kövér L Kardos G. The rise of Antimicrobial resistance in Wild birds: Potential AMR sources and Wild birds as AMR reservoirs and disseminators : literature review. Magyar Állatorvosok Lapja. (2024) 146:91–105. doi: 10.56385/magyallorv.2024.02.91-105

[11] Boyce JM Opal SM Chow JW Zervos MJ Potter-Bynoe G Sherman CB . Outbreak of multidrug-resistant *Enterococcus faecium* with transferable vanB class vancomycin resistance. J Clin Microbiol. (1994) 32:1148–53. doi: 10.1128/jcm.32.5.1148-1153.1994 8051238 PMC263627

[12] McKessar SJ Berry AM Bell JM Turnidge JD Paton JC. Genetic Characterization of vanG, a Novel Vancomycin Resistance Locus of *Enterococcus faecalis*. Antimicrob Agents Chemother. (2000) 44:3224–8. 11036060 10.1128/aac.44.11.3224-3228.2000 PMC101640

[13] Berktas M Yaman G Ozturk O. vanC Gene-Related Intrinsic Teicoplanin Resistance Detected in *Enterococcus casseliflavus* and *E.* gallinarum Strains by the BD Phoenix Automated Microbiology System. J Clin Microbiol. (2008) 46:2466–2466. doi: 10.1128/JCM.00795-08 18463205 PMC2446911

[14] Sletvold H Johnsen PJ Wikmark O-G Simonsen GS Sundsfjord A Nielsen KM. Tn1546 is part of a larger plasmid-encoded genetic unit horizontally disseminated among clonal *Enterococcus faecium* lineages. J Antimicrob Chemother. (2010) 65:1894–906. doi: 10.1093/jac/dkq219 20558469 PMC2920175

[15] Ghidán A Kaszanyitzky EJ Dobay O Nagy K Amyes SGB Rozgonyi F. Distribution and genetic relatedness of vancomycin-resistant enterococci (VRE) isolated from healthy slaughtered chickens in Hungary from 2001 to 2004. Acta Vet Hung. (2008) 56:13–25. doi: 10.1556/avet.56.2008.1.3 18401953

[16] Lorincz EÉ Wagenhoffer Z Zenke P. The relevance of epigenetic research in veterinary sciences: Literature review. Magyar Állatorvosok Lapja. (2025) 147:429–41.

[17] Rogers LA Strong K Cork SC McAllister TA Liljebjelke K Zaheer R . The Role of Whole Genome Sequencing in the Surveillance of Antimicrobial Resistant Enterococcus spp: A Scoping Review. Front Public Health. (2021) 9:599285. doi: 10.3389/fpubh.2021.599285 34178909 PMC8222819

[18] Muloi DM Jauneikaite E Anjum MF Essack SY Singleton DA Kasudi MR . Exploiting genomics for antimicrobial resistance surveillance at One Health interfaces. Lancet Microbe. (2023) 4:e1056–62. doi: 10.1016/S2666-5247(23)00284-7 37977165

[19] Aqib AI Ijaz M Farooqi SH Ahmed R Shoaib M Ali MM . Emerging discrepancies in conventional and molecular epidemiology of methicillin resistant *Staphylococcus aureus* isolated from bovine milk. Microb Pathog. (2018) 116:38–43. doi: 10.1016/j.micpath.2018.01.005 29325865

[20] Rasheed H Ijaz M Ahmed A Javed MU Shah SFA Anwaar F. Discrepancies between phenotypic and genotypic identification methods of antibiotic resistant genes harboring *Staphylococcus aureus*. Microb Pathog. (2023) 184:106342. doi: 10.1016/j.micpath.2023.106342 37704062

[21] Muzzey D Evans EA Lieber C. Understanding the Basics of NGS: From Mechanism to Variant Calling. Curr Genet Med Rep. (2015) 3:158–65. doi: 10.1007/s40142-015-0076-8 26566462 PMC4633438

[22] Mihály Z Gyorffy B. Next generation sequencing technologies (NGST) development and applications. Orv Hetil. (2011) 152:55–62. doi: 10.1556/OH.2011.29007 21177232

[23] Johansson MHK Bortolaia V Tansirichaiya S Aarestrup FM Roberts AP Petersen TN. Detection of mobile genetic elements associated with antibiotic resistance in *Salmonella enterica* using a newly developed web tool: MobileElementFinder. J Antimicrob Chemother. (2021) 76:101–9. doi: 10.1093/jac/dkaa390 33009809 PMC7729385

[24] Hammerum AM. Enterococci of animal origin and their significance for public health. Clin Microbiol Infect. (2012) 18:619–25. doi: 10.1111/j.1469-0691.2012.03829.x 22487203

[25] Park IJ Lee WG Shin JH Lee KW Woo GJ. VanB Phenotype-vanA Genotype *Enterococcus faecium* with Heterogeneous Expression of Teicoplanin Resistance. J Clin Microbiol. (2008) 46:3091–3. doi: 10.1128/JCM.00712-08 18596139 PMC2546774

[26] Kerek Á Szabó Á Jerzsele Á. Antimicrobial Susceptibility Profiles of Commensal *Enterococcus* spp. Isolates from Chickens in Hungarian Poultry Farms Between 2022 and 2023. Antibiotics. (2024) 13:1194. doi: 10.3390/antibiotics13121194 39766584 PMC11672767

[27] Kerek Á Szabó Á Barnácz F Csirmaz B Kovács L Jerzsele Á. Antimicrobial Susceptibility Profiles of Commensal *Enterococcus* spp. Isolates from Turkeys in Hungarian Poultry Farms Between 2022 and 2023. Antibiotics. (2025) 14:331. doi: 10.3390/antibiotics14040331 40298473 PMC12024081

[28] Caddey B Shaukat W Tang KL Barkema HW. Vancomycin-resistant *Enterococcus* prevalence and its association along the food chain: a systematic review and meta-analysis. J Antimicrob Chemother. (2025) 80:908–18. 39849844 10.1093/jac/dkaf008 PMC11962378

[29] Papp M Solymosi N. Review and Comparison of Antimicrobial Resistance Gene Databases. Antibiotics. (2022) 11:339. doi: 10.3390/antibiotics11030339 35326803 PMC8944830

[30] Skoulakis A Kostoula V Daniilidis K Gkountinoudis C-G Petinaki E Hatzigeorgiou AG. AmrProfiler: a comprehensive tool for identifying antimicrobial resistance genes and mutations across species. Nucleic Acids Res. (2025) 53:W20–31. doi: 10.1093/nar/gkaf400 40347103 PMC12230725

[31] Hawkins MR Medvedeva N Wang H Banaei N Holubar MK. “Keeping us on our toes”: a review of what clinicians need to know about vancomycin-variable *Enterococcus*. Antimicrob Steward Healthc Epidemiol. (2024) 4:e200. doi: 10.1017/ash.2024.449 39563924 PMC11574585

[32] Afshari A Taheri S Hashemi M Norouzy A Nematy M Mohamadi S. Methicillin- and Vancomycin-Resistant *Staphylococcus aureus* and Vancomycin-Resistant Enterococci Isolated from Hospital Foods: Prevalence and Antimicrobial Resistance Patterns. Curr Microbiol. (2022) 79:326. doi: 10.1007/s00284-022-03022-0 36125553

